# Neoadjuvant ipilimumab (3 mg/kg or 10 mg/kg) and high dose IFN-α2b in locally/regionally advanced melanoma: safety, efficacy and impact on T-cell repertoire

**DOI:** 10.1186/s40425-018-0428-5

**Published:** 2018-10-23

**Authors:** Ahmad Tarhini, Yan Lin, Huang Lin, Zahra Rahman, Priyanka Vallabhaneni, Prateek Mendiratta, James F. Pingpank, Matthew P. Holtzman, Erik C. Yusko, Julie A. Rytlewski, Uma N. M. Rao, Robert L. Ferris, John M. Kirkwood

**Affiliations:** 1 UPMC Hillman Cancer Center, Pittsburgh, USA; 2 Department of Hematology and Oncology, Cleveland Clinic Taussig Cancer Institute and Case Comprehensive Cancer Center, 9500 Euclid Ave CA6-157, Cleveland, OH 44195 USA; 3 Adaptive Biotechnologies, Seattle, USA

**Keywords:** Immunotherapy, Ipilimumab, Anti-CTLA-4, Interferon, Melanoma

## Abstract

**Background:**

Neoadjuvant immunotherapy utilizing novel combinations has the potential to transform the standard of care for locally/regionally advanced melanoma. We hypothesized that neoadjuvant ipilimumab in combination with high dose IFNα2b (HDI) is safe and associated with durable pathologic complete responses (pCR).

**Methods:**

Patients with locally/regionally advanced melanoma were randomized to ipilimumab 3 or 10 mg/kg × 4 doses bracketing definitive surgery, then every 12 weeks × 4. HDI was given concurrently. We evaluated the safety and efficacy of the combination with ipilimumab 3 or 10 mg/kg. The impact on T-cell fraction and clonality were investigated in tumor and blood.

**Results:**

Thirty patients (age 37–76), 15 each at 3 and 10 mg/kg, 18 male and 12 female were treated. Considering immune related adverse events (irAEs) of interest, more grade 3/4 irAEs were seen with ipilimumab 10 mg/kg versus 3 mg/kg (*p* = 0.042). Among 28 evaluable patients, 11 relapsed, of whom 5 died. Median follow-up for 17 patients who have not relapsed was 32 months. The radiologic preoperative response rate was 36% (95% CI, 21–54); 4 patients at ipilimumab 3 mg/kg and 6 at 10 mg/kg and 2 (at 10 mg/kg) later relapsed. The pCR was 32% (95% CI, 18–51); 5 patients at ipilimumab 3 mg/kg and 4 at 10 mg/kg and one (at 3 mg/kg) had a late relapse. In patients with pCR, T-cell fraction was significantly higher when measured in primary melanoma tumors (*p* = 0.033). Higher tumor T-cell clonality in primary tumor and more so following neoadjuvant therapy was significantly associated with improved relapse free survival.

**Conclusions:**

Neoadjuvant ipilimumab-HDI was relatively safe and exhibited promising tumor response rates with an associated measurable impact on T-cell fraction and clonality. Most pCRs were durable supporting the value of pCR as a primary endpoint in neoadjuvant immunotherapy trials.

**Trial registration:**

ClinicalTrials.gov, NCT01608594. Registered 31 May 2012.

**Electronic supplementary material:**

The online version of this article (10.1186/s40425-018-0428-5) contains supplementary material, which is available to authorized users.

## Background

Patients with melanoma and clinically detectable regional lymphatic metastases have an unacceptable high 5-year relapse rate approaching 70–90% [1]. Local or regional relapse after initial surgical management portends a similar poor prognosis [2, 3]. The 5 and 10 year survival rates were 9–11% and 5%, respectively after a local recurrence in the Melanoma Intergroup Surgical Trial [3]. The current standard of care consists of aggressive surgical management aimed at rendering the patient disease free surgically, followed by systemic adjuvant therapy [4–7]. For these patients, there continues to be an urgent need to improve the clinical outcome through novel systemic neoadjuvant approaches that may minimize the surgical intervention in an era of unprecedented advances in systemic therapy [8].

Patients with advanced melanoma display strong Th2-type polarization [9, 10]. Both CTLA-4 blockade and IFNα can up-regulate the pro-inflammatory cytokine response (Th1 polarization) [11, 12], and are associated with increased T-cell and dendritic cell (DC) tumor infiltration [13–15]. The impact of IFNα on DCs is well established, affecting stages of myeloid DC generation, maturation, differentiation and function [16]. In immature states, IFN-treated DCs induce a ‘polarized’ Th1 cytokine microenvironment [17]. Similar to myeloid DCs, IFNs polarize lymphocytes toward a pro-inflammatory Th1phenotype [18–20]. In the cytotoxic T-cell compartment, type I IFNs induce antitumor cell-mediated cytotoxicity [21], and promote natural killer (NK) cell-mediated proliferation and cytotoxicity [22]. This Th1 shift in immunity induced by IFNα can be countered by other mechanisms (e.g. CTLA-4) explaining very limited activity observed with IFNα as monotherapy in metastatic melanoma. Combining IFNα with CTLA-4 blockade may, however, alter this balance by down-regulating the CTLA4 suppressive regulatory elements.

We previously reported the results of a phase II study of the combination of IFNα-2b and the anti-CTLA4 antibody tremelimumab in patients with advanced inoperable melanoma [23]. The best durable response rate by intent to treat analysis of that trial (*N* = 37) was 24%. Median progression-free survival (PFS) was 6.4 months (95% CI = 3.3, 13.1). Median overall survival (OS) was 21 months (95% CI = 9.5, −) [23]. In that trial, baseline absolute lymphocyte count (ALC) of ≥1000/uL was associated with both response (*p* = 0.02) and disease control (*p* = 0.03) [24]. These data supported further investigation of the combination of IFNα and CTLA4 blockade.

The clinical development of ipilimumab led to regulatory approval of the lower dose regimen (3 mg/kg) for the treatment of advanced inoperable melanoma, based on OS benefit in a landmark phase III trial [25], although a higher dosage regimen (10 mg/kg) was approved in the adjuvant setting [26]. An early phase II dose-ranging study suggested a small survival advantage for the higher dosage in terms of clinical activity, at the expense of significantly increased toxicity [27], that was further supported by a recently reported phase III trial [28]. Therefore, we conducted a randomized trial to evaluate the safety profile and efficacy of combination immunotherapy with high dose IFNα -2b (HDI) and ipilimumab at 3 mg/kg or 10 mg/kg, as a neoadjuvant therapy for locally/regionally advanced operable melanoma. Endpoints included safety, pathological response rate, radiologic preoperative response rate and the impact on T-cell fraction and clonality in tumor and peripheral blood.

## Methods

### Patients

Eligible patients were at least 18 years old and presented with clinically detectable locally and/or regionally advanced melanoma (cutaneous, mucosal or unknown primary) including (1) Tx or T1–4 and (2) N1b, or N2b, or N2c, or N3 and (3) M0 (AJCC 7th edition). Patients were required to meet protocol specified safety and tumor measurability criteria. Prior treatment with adjuvant HDI was allowed. The study was initiated after approval from the institutional review board (IRB) and was conducted in accordance with the Declaration of Helsinki. A University of Pittsburgh IRB approved written informed consent (IRB# PRO12020161) was obtained from all patients.

### Study design and treatment

The phase I study was designed as a randomized safety (primary endpoint) and efficacy (secondary) study. Patients were randomized to receive ipilimumab intravenously (IV) at 3 mg/kg or 10 mg/kg every 3 weeks for 2 doses followed by definitive surgery (6–8 weeks after initiation of systemic therapy). After recovery from surgery and any adverse events (AEs), treatment with ipilimumab was resumed at the same dosage for 2 additional doses given 3 weeks apart. Ipilimumab was then continued in the absence of limiting AEs for up to 4 additional doses given 12 weeks apart. HDI was given concurrently IV at 20 MU/m^2^/day, 5 days/week for 4 weeks followed by 10 MU/m^2^/day subcutaneously (SC) every other day, 3 days/week for 2 weeks prior to definitive surgery. After recovery from surgery and limiting AEs, treatment with HDI was resumed at the same SC regimen dosing for 46 additional weeks.

When available, samples of the primary tumor biopsies were collected. A pre-treatment metastatic tumor biopsy was obtained followed by a second biopsy at definitive surgery. Blood specimens were also collected at baseline, 6 weeks, then at 3, 6 and 12 months. Blood was drawn into heparin (for peripheral blood mononuclear cells; PBMC) tubes or tubes without anticoagulant (serum) and processed by the Immunologic Monitoring Lab upon receipt. Tumor biopsies were transported to the Tissue Procurement Facility after surgery in sterile medium. Part of the tumor was formalin-fixed and paraffin-embedded, and part was enzymatically digested to single cells and cryopreserved. When available, samples of the relapse tumor biopsies were collected.

### Safety and efficacy assessments

The descriptions and grading scales of the NCI Common Terminology Criteria for Adverse Events (CTCAE) version 4.0 were used for adverse event grading and reporting. Toxicity specific management guidelines were provided separately in the study protocol for both ipilimumab and HDI. Patients were allowed to continue ipilimumab or HDI if a limiting AE was determined to be related to one agent but not the other. For the purpose of tumor response evaluation (modified WHO criteria), imaging studies were conducted at baseline, 6–8 weeks after initiating systemic therapy (before surgery) and then every 3 months. Responses were designated as complete (CR), partial (PR), stable disease (SD) or disease progression (PD). Due to the planned definitive surgery, it was not possible to confirm the responses radiologically. Pathologic complete response (pCR) was assessed at the time of definitive surgery and was defined as no visible or viable malignant cells on haematoxylin and eosin staining during histologic assessment by a surgical pathologist (U.N.M.R.). Further, if only single cancer cells or minute clumps of cancer cells were observed on histologic assessment after definitive surgery, such patients were classified as having a Microscopic residual disease (MRD) according to the reporting pathologist.

### Statistical analysis

The response rates were estimated by the percentage of patients who achieved a pCR as well as clinical/radiologic CR, PR, or SD (by mWHO criteria) with corresponding 90% confidence limits. The Kaplan-Meier estimates of PFS and OS, with corresponding median survival (95% confidence limits) were determined.

### Laboratory methods and corresponding statistical analyses

#### Biospecimen processing

The biopsy samples of tumor were formalin fixed and paraffin-embedded. For PBMC, blood was drawn into heparin tubes and for serum into tubes without anticoagulant and processed by the Immunologic Monitoring Lab upon receipt at baseline and at 6 weeks. PBMC were isolated by Ficoll gradient and cryopreserved for batched testing according to standard operating procedures. The sample freezers were monitored carefully for temperature fluctuations, and maintained the samples at -80C.

#### T-cell receptor sequencing

Tumor biopsies of metastatic melanoma were evaluable for testing at pretreatment (before initiating study therapy; *N* = 20 patients), definitive surgery (week 6–8; *N* = 25) and melanoma relapse (*N* = 6). In addition, specimens of primary melanoma were available (*N* = 24). PBMC were available for testing at pretreatment (*N* = 29), 6 weeks following the initiation of ipilimumab (*N* = 24), then at 3 (*N* = 23), 6 (*N* = 21) and 12 (*N* = 14) months.

Immunosequencing of CDR3 of human TCRβ chains was performed using the immunoSEQ® Assay (Adaptive Biotechnologies, Seattle, WA). Extracted genomic DNA was amplified in a bias-controlled multiplex PCR, followed by high-throughput sequencing. Sequences were collapsed and filtered to identify and quantitate the absolute abundance of each unique TCRβ CDR3 for further analysis, as previously described [29–31]. Immunosequencing was performed to determine TCRB clonality and T-cell fraction (fraction of T cells within the total nucleated cell count) in the tumor microenvironment (TME) and PBMC tissue compartments. The clonality metric quantitates the extent of mono- or oligo-clonal expansion by measuring the shape of the clone frequency distribution. Values range from 0 to 1, where values approaching 1 indicate a nearly monoclonal population. The fraction of T-cells in tissue samples was calculated by normalizing TCRβ template counts [30] to the total amount of DNA usable for TCR sequencing [31], where the amount of usable DNA was determined by PCR-amplification and sequencing of several reference genes that are expected to be present in all nucleated cells.

## Results

### Patient characteristics

The baseline patient demographics and disease characteristics are summarized in Table 1.

**Table 1:** Patient demographics and baseline disease characteristics (*N* = 30 patients)

Variable	No. of patients (%)
Age (years; Median (range)	61 (37–76)
Cutaneous primary	21 (70)
Unknown primary	8 (27)
Mucosal	1 (3)
Gender	
Female	12 (40)
Male	18 (60)
Performance status	
0	16 (53)
1	14 (47)
Recurrent disease after prior surgery	15 (50)
Presence of in-transit metastases	16 (53)
Prior adjuvant HDI	5 (17)
Estimated risk Stage	
IIIB	3 (10)
IIIC	25 (83)
IV (Not eligible)	2 (7)

### Treatment details

Over 120 cycles of ipilimumab therapy were delivered to patients on this study, with a median of four cycles per patient. HDI was given in combination during these cycles and managed in accordance to the HDI dose holding and discontinuation criteria. Among 30 patients treated, the attrition rate for HDI was 9 (PD) and 6 (AE), while 15 patients completed the planned course of treatment.

### Safety

The toxicities observed were consistent with the known AE profiles of ipilimumab and HDI. Table 2 summarizes by severity the adverse events (AEs) that were considered related to the study regimen. Among listed toxicities including, fever, fatigue, creatinine increase, neutropenia, thrombocytopenia and isolated nausea and vomiting (in the absence of autoimmune entero-colitis), all were reversible with holding HDI dosing and without the need for corticosteroids supporting HDI as a cause rather than ipilimumab. Table 3 summarizes grade 3–4 immune related AEs by study arm. Among irAEs of interest summarized in Table 3, more grade 3/4 irAEs were seen among recipients of ipilimumab at 10 mg/kg compared to ipilimumab at 3 mg/kg (*p* = 0.042). There were no delays in the planned definitive surgery secondary to ongoing toxicity resulting from the neoadjuvant induction phase. One patient (ipilimumab 10 mg/kg) with a posterior trunk primary melanoma was found to have disease progression in a separate nodal site at imaging preoperatively. The decision was made to treat for 6 additional weeks after which he experienced a significant radiologic response and was rendered disease free surgically.

**Table 2:** Adverse events (worst grade; ≥ 2pts) possibly, probably or definitely related to the study regimen (*N* = 30)

Type	Any Grade		Grade 3		Grade 4	
	No. patients	%	No. Pts.	%	No. Pts.	%
Skin						
Pruritus	19	63	2	7	0	0
Rash maculopapular	14	47	6	20	0	0
Gastrointestinal						
Diarrhea/Colitis	15	50	3	10	0	0
Nausea	16	53	1	3	0	0
Vomiting	9	30	1	3	0	0
Hepatic						
ALT	22	73	6	20	0	0
AST	28	93	5	17	1	3
Endocrine						
Hypothyroidism	8	27	0	0	0	0
Hyperthyroidism	3	10	0	0	0	0
Adrenal insufficiency	10	33	3	10	0	0
Hypophysitis	2	7	2	7	0	0
Lipase increased	9	30	3	10	0	0
Hematologic						
Neutropenia	21	70	2	7	0	0
Thrombocytopenia	20	67	0	0	0	0
Other						
Fever	17	57	0	0	0	0
Fatigue	27	90	16	53	0	0
CPK increased	19	63	2	7	0	0
Creatinine increased	6	20	2	7	0	0

**Table 3:** Grade 3–4 immune related adverse events divided by study arm (*N* = 30 patients)

	Any Grade		Grade 3		Grade 4		Grade 3/4 Ipi 3 mg/kg		Grade 3/4 Ipi 10 mg/kg	
	No. Pts.	%	No. Pts.	%	No. Pts.	%	No. Pts.	%	No. Pts.	%
Adrenal insufficiency	10	33	2	7	0	0	0	0	2	7
Hypophysitis	2	7	2	7	0	0	0	0	2	7
Diarrhea/Colitis	15	50	3	10	0	0	2	7	1	3
AST/ALT	28	93	5	17	1	3	2	7	4	13
Lipase increased	9	30	3	10	0	0	0	0	3	10
Rash, maculo-papular	14	47	7	23	0	0	4	13	3	10
Pneumonitis	1	3	1	3	0	0	0	0	1	3
Autoimmune nephritis	1	3	1	3	0	0	0	0	1	3

### Efficacy analysis

Among 28 evaluable patients, 11 relapsed, of whom 5 died. The median follow-up for the 17 patients who have not relapsed is 32 months. Response rates by histological and radiological assessments are summarized in Table 4 by study arm. In addition to patients who achieved a pCR, 2 additional patients had only microscopic residual disease (MRD) at definitive surgery. Among patients with pCR or MRD (*N* = 11) one with pCR (at 3 mg/kg) experienced isolated nodal relapse after 18 months and was rendered disease free surgically. Therefore, after a median follow up of 32 months, 10/11 patients with pCR or MRD continued to be disease free. Among patients with clinical and radiologic response, 7/10 showed pCR and 1/10 had MRD. Further, 2 patients with clinical and radiologic response relapsed after 7 and 11 months, respectively. Both of these patients had gross residual disease at the time of definitive surgery. Therefore, at a median follow up interval of 32 months, 8/10 radiologic responders continued to be disease free.

**Table 4:** Response rate (RR) by radiologic (preoperative; 6 weeks from baseline) and histologic (6–8 weeks from baseline) assessments

	All patients (*N* = 28)		Ipi 3 mg/kg (n = 14)		Ipi 10 mg/kg (n = 14)	
	No. pts.	%	No. pts.	%	No. pts.	%
Radiologic preoperative RR (WHO; unconfirmed)	10 (9 PR, 1CR)	36%	4 (3 PR, 1 CR)	29	6 (All PR)	43
Pathologic complete RR (no viable tumor on histologic assessment)	9	32%	5	36	4	29
Among radiologic responders						
• One relapsed and later responded to anti-PD1 antibody therapy						
Among complete pathologic responders						
• None relapsed to date						

Median PFS was not reached. The probability of PFS at 6 and 12 months was 0.86, 95% CI (0.74, 1.0) and 0.79, 95% CI (0.65, 0.95) respectively. The probability of OS at 12 and 24 months was 0.93, 95% CI (0.84, 1.0) and 0.89, 95% CI (0.79, 1.0).

### Immune correlates

**Fig. 1:**
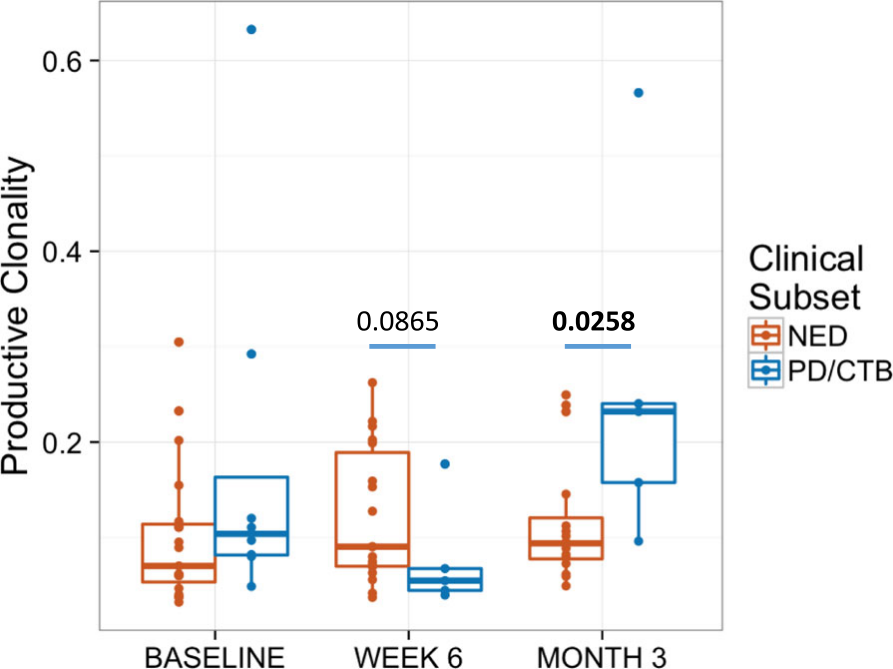
Peripheral blood mononuclear cells (PBMC) T-Cell Clonality in patients with no evidence of disease relapse after surgery (durable NED) versus patients with disease progression with or without subsequent death (PD/CTB). Measurements were made in PBMC at baseline (before the initiation of systemic therapy), then at 6 weeks and 3 months

**Fig. 2:**
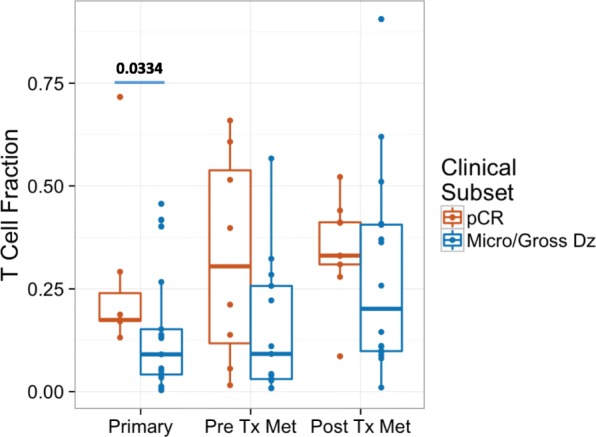
Tumor T-Cell fraction in patients with pathologic complete response (pCR) versus residual tumor at the time of definitive surgery (approximately 6–8 weeks from baseline). Measurements were made in primary melanoma tumor biopsies (Primary), pre-treatment metastatic melanoma biopsies (Pre Tx Met) and post-treatment metastatic melanoma biopsies (Post Tx Met; approximately 6–8 weeks from baseline)

**Fig. 3:**
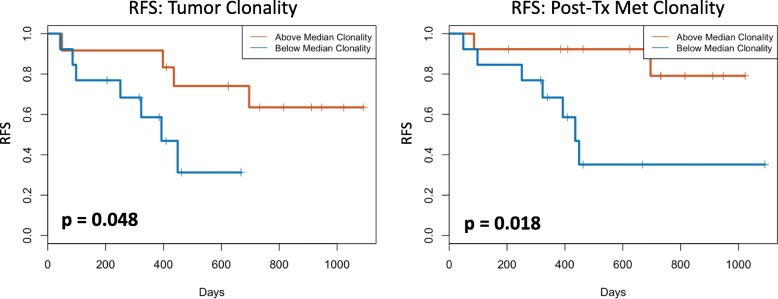
Tumor infiltrating lymphocyte (TIL) clonality in primary melanoma tumor biopsies (Primary Tumor) and post-treatment metastatic melanoma biopsies (Post Tx Met; approximately 6–8 weeks from baseline). Higher TIL clonality in primary tumor, and more so following neoadjuvant immunotherapy, was associated with improved relapse free survival (RFS)

PBMC clonality trended towards lower values at baseline and became significantly lower at 12 weeks (*p* = 0.025) for patients who continued to be relapse free (NED) versus those who relapsed following definitive surgery, as shown in Fig. 1. In the TME, among patients with pCR the tumor infiltrating lymphocyte (TIL) fraction was significantly higher when measured in primary melanoma tumors (*p* = 0.033) and tended to be higher throughout at other time points as shown in Fig. 2. No significant impact upon T-cell fraction in the TME was seen when comparing the two different ipilimumab dosages. Further, higher TIL clonality in the primary tumor, and following neoadjuvant immunotherapy (~ 6–8 weeks from baseline) was found to be associated with improved relapse-free survival, Fig. 3. The number of tumor-associated clones in the baseline tumor biopsies that were expanded in the blood following neoadjuvant treatment was strongly correlated with both TIL fraction (Rho 0.7299, *p* = 0.0003) and TIL clone diversity (Rho 0.882, *p* = 2.7^− 7^), Additional file 1: Figure S4.

## Discussion

This tested regimen utilizing CTLA4 blockade in combination with IFNα provides a major advance over neoadjuvant ipilimumab monotherapy as we had previously tested [32]. In this study, the clinical and radiological preoperative response rate to neoadjuvant therapy with IFN + Ipi prior to definitive surgery (WHO; unconfirmed) was 36% (10/28) and the pCR rate in the definitive surgical specimen (no viable tumor on histologic assessment) was 32% (9/28), without significant differences between the two ipilimumab dosages of 3 mg/kg and 10 mg/kg. Among patients with pCR or MRD (*N* = 11) one with pCR experienced a late isolated nodal relapse and was rendered disease free surgically. Therefore, after a median follow up of 32 months, 10/11 patients with pCR or MRD continued to be disease free. Among patients with clinical and radiologic response, 7/10 showed pCR and 1/10 had MRD. Further, at a median follow up interval of 32 months, 8/10 radiologic responders continued to be disease free. These data support the value of pCR as a primary endpoint in future neoadjuvant immunotherapy trials, as a meaningful clinical endpoint of therapy that correlates with durable antitumor benefits. It is also encouraging that there is a strong correlation between preoperative clinical and radiologic responses and pCR and pathologically confirmed MRD. Ultimately, with the utilization of novel radiologic imaging and radiolabelling techniques, it would be important to attempt radiological prediction of major histologic responses and durable clinical benefits, in the absence of additional surgery. Such efforts are being adopted in the next generation of neoadjuvant studies in the national clinical trials network (EA6182; neoadjuvant anti-PD1 based immunotherapy combinations in melanoma). Current optimal imaging and radiomics techniques that will take advantage of advanced molecular imaging and high-throughput computing programs are an area of active investigation, with promise in this regard [33].

For patients with locally-regionally advanced melanoma, neoadjuvant therapy utilizing novel combinations of immunotherapeutic and targeted agents has the potential to transform the standard of care, and to guide the selection of new regimens for phase III trial evaluation. Neoadjuvant therapy has supported the ability to improve clinical outcome of many solid tumors including breast, head and neck, bladder, esophageal and rectal cancers [34–37]. Improvements in survival, surgical resectability, local control and organ preservation are now more likely with new effective agents available for the treatment of melanoma [34–37]. The ability to evaluate and compare pathologic and clinical/radiologic responses are of inestimable value to the field, where the conduct of formal phase III trials to evaluate current combinations otherwise exceeds the capacity of the system. In breast cancer, pCR after neoadjuvant chemotherapy has been shown to be significantly associated with favorable long-term survival rates [38–40]. This has led to the recommendation by the Food and Drug Administration (FDA) that pCR be evaluated as a primary end point in neoadjuvant breast cancer trials. Compared with chemotherapy, objective complete responses with immunotherapy in advanced melanoma are more likely to correlate with durable disease control and survival benefits, indirectly supporting the value of pCR in neoadjuvant melanoma immunotherapy trials [41–43].

Previous neoadjuvant studies in melanoma have tested chemotherapy with temozolomide given preoperatively orally in 2 cycles, where limited clinical activity and rare objective responses without pCR were noted [44]. Neoadjuvant biochemotherapy (BCT) with cisplatin, vinblastine, dacarbazine, interleukin-2 and IFNα was tested in 2 studies [45, 46]. Response rates approaching 40–50% and pCR rates of 6–11% were reported. However, following the results of RCTs in metastatic disease showing the lack of survival improvement over chemotherapy, BCT has generally been abandoned [41]. Neoadjuvant immunotherapy studies in melanoma to date have included trials of HDI, ipilimumab at 10 mg/kg, and the combinations of nivolumab with ipilimumab; targeted approaches with dabrafenib and trametinib have also been reported [47–49]. Among 20 patients treated with neoadjuvant HDI, 3 had pCR and the overall clinical response rate was 55% [50]. In the neoadjuvant study of ipilimumab (*N* = 33), no pCR was found and the clinical response rate was ~ 10% [32]. These studies yielded evidence of promising clinical activity, but have, moreover, shown important biomarker and biological findings that illuminated the mechanism of action for these agents [32, 51]. Neoadjuvant studies of other molecularly targeted and immunotherapeutic agents are ongoing [52]. A recently reported study tested neoadjuvant dabrafenib and trametinib in BRAF-mutant melanoma. Among 13 evaluable patients treated with the combination, 11 (85%) achieved an objective response (2 CR, 9 PR). Among 12 patients who underwent surgery, 7 (58%) achieved pCR. Interestingly, event-free survival after surgery did not significantly differ by pathological response, but patients with a pCR had a significantly longer distant metastasis-free survival [53].

In the present study, the toxicity profile observed in the neoadjuvant setting was consistent with what is known for HDI and for ipilimumab, in terms of the quality and frequency of adverse events. The rates of immune related AEs (irAE) were also similar to reported rates with ipilimumab at 3 mg/kg or 10 mg/kg [54], with less frequent irAEs seen at 3 mg/kg. Our findings are consistent with a recent phase 3 trial testing the benefit-risk profile of ipilimumab 3 mg/kg versus 10 mg/kg, showing increased treatment-related adverse events with 10 mg/kg, with modest improvement in OS that did not lead to labeling or practice changes [28]. Further, we recently reported an interim analysis of E1609 adjuvant trial evaluating the safety and RFS of concurrently randomized high-risk melanoma patients who received ipilimumab at either 10 mg/kg or 3 mg/kg. Our analysis found significantly more toxicity with the higher dosage, but no difference in RFS at a median follow up interval of 3.1 years [55].

In the present neoadjuvant study, we immunosequenced the TCR in the TME and PBMC to evaluate the repertoire of T-cell clonality and diversity and T-cell fraction at baseline and following 6 weeks of neoadjuvant therapy. The TIL fraction in the TME or primary tumors was significantly correlated with pCR during neoadjuvant therapy. Further, PBMC clonality expanded following treatment, in parallel to TIL clonality expansion in the TME, which correlated with improved clinical outcome. Therefore, the impact of this systemic neoadjuvant immunotherapy is evident both systemically in the circulation and locally in the TME. Interestingly, patients with higher TIL fractions in the TME were more likely to have tumor-associated clones detectable in the blood, suggesting that these clones may be useful for tracking the immune response. Local and systemic T-cell clonal expansion has been positively correlated with clinical outcomes after immunotherapy in melanoma and other solid tumors [56–59]. In metastatic melanoma, patients treated with anti-PD1 immunotherapy, TIL clonality increased by more than ten times in patients with antitumor response compared to those who progressed [56]. In a small cohort of 12 patients treated with ipilimumab alone, TCR diversity in the peripheral blood was associated with clinical outcomes [57]. A study of PBMCs from patients with castration resistant prostate cancer and melanoma treated with ipilimumab or tremelimumab suggested that CTLA-4 blockade leads to T-cell repertoire evolution and diversification—and that improved clinical outcomes were associated with preservation of clonotype diversity [58].

Recent landmark adjuvant studies have shown dramatic improvement in the relapse free survival of resected stage III melanoma with the use of single agent nivolumab or single agent pembrolizumab or with the use of dual targeted therapy (dabrafenib and trametinib) for BRAF mutant melanoma [60–62]. Neoadjuvant therapy for locally-regionally advanced melanoma is an area of active investigation that also appears to be destined to change the current clinical practice in managing this disease, taking advantage of emerging active agents and combinations. Therefore, this regimen could be proposed for phase III testing if current studies appear less effective. If taken forward, we would utilize the low ipilimumab dose regimen.

## Conclusions

Neoadjuvant immunotherapy with ipilimumab and HDI for patients with locally-regionally advanced melanoma exhibits promising pCR and radiologic response rates at both 3 and 10 mg/kg dosage levels of ipilimumab. Most responses were durable, supporting the value of pCR as a primary endpoint in neoadjuvant immunotherapy trials.

## Additional file

Additional file 1: **Figure S4.** Clonal relatedness in the tumor and blood. The number of tumor-associated clones in the baseline tumor biopsies that were expanded in blood post-treatment was strongly correlated with both (A) tumor infiltrating lymphocyte (TIL) fraction (Rho 0.7299, *p* = 0.0003) and (B) TIL clone diversity (Rho 0.882, *p* = 2.7^− 7^). (PPTX 338 kb)
