# Supplementary figures and images for: Neoadjuvant ipilimumab (3 mg/kg or 10 mg/kg) and high dose IFN-α2b in locally/regionally advanced melanoma: safety, efficacy and impact on T-cell repertoire
Source: J Immunother Cancer. 2018 Oct 23;6:112. doi: 10.1186/s40425-018-0428-5 (PMC6199801; doi:10.1186/s40425-018-0428-5)

## Slide 1
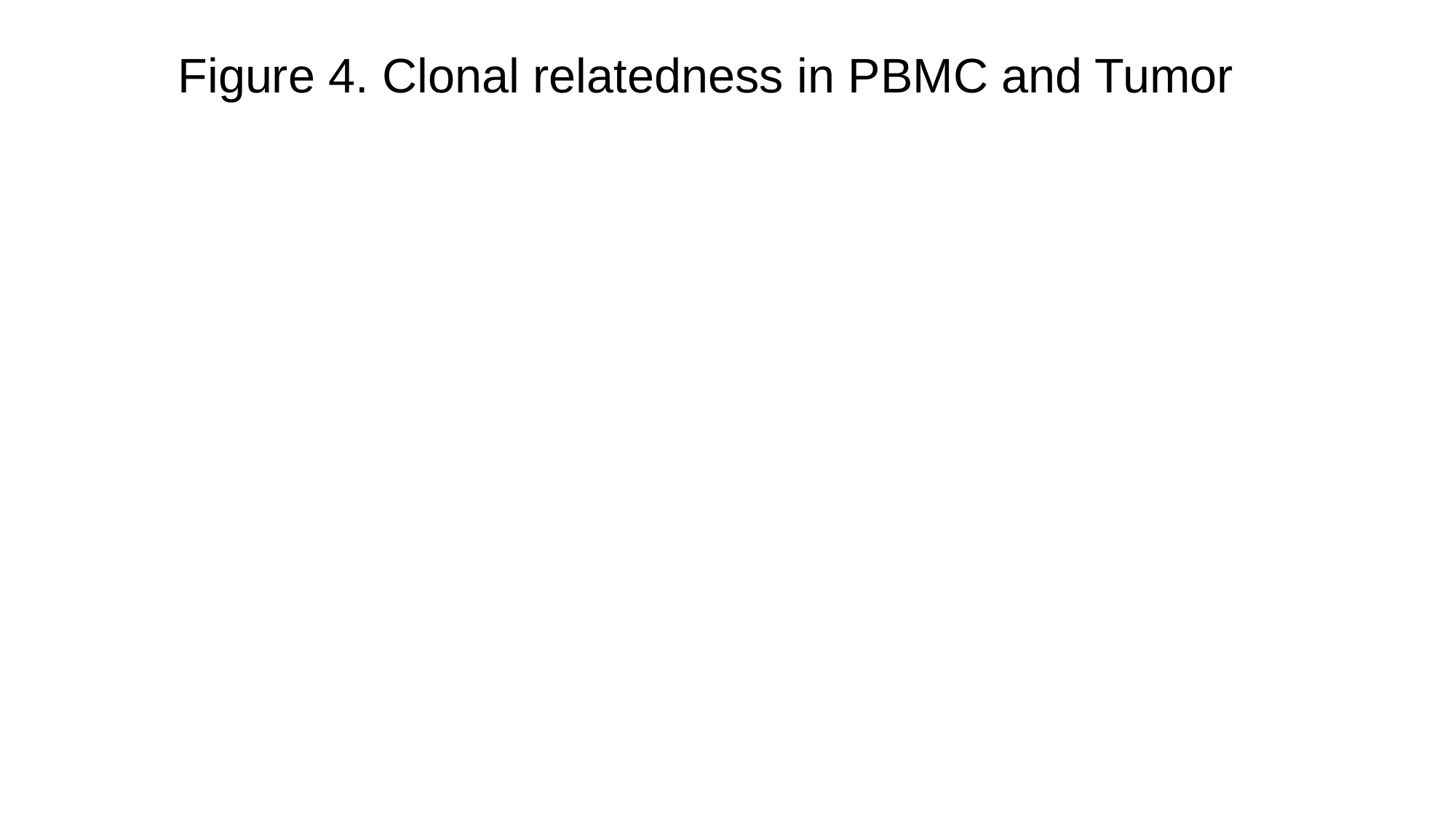

Figure 4. Clonal relatedness in PBMC and Tumor

## Slide 2
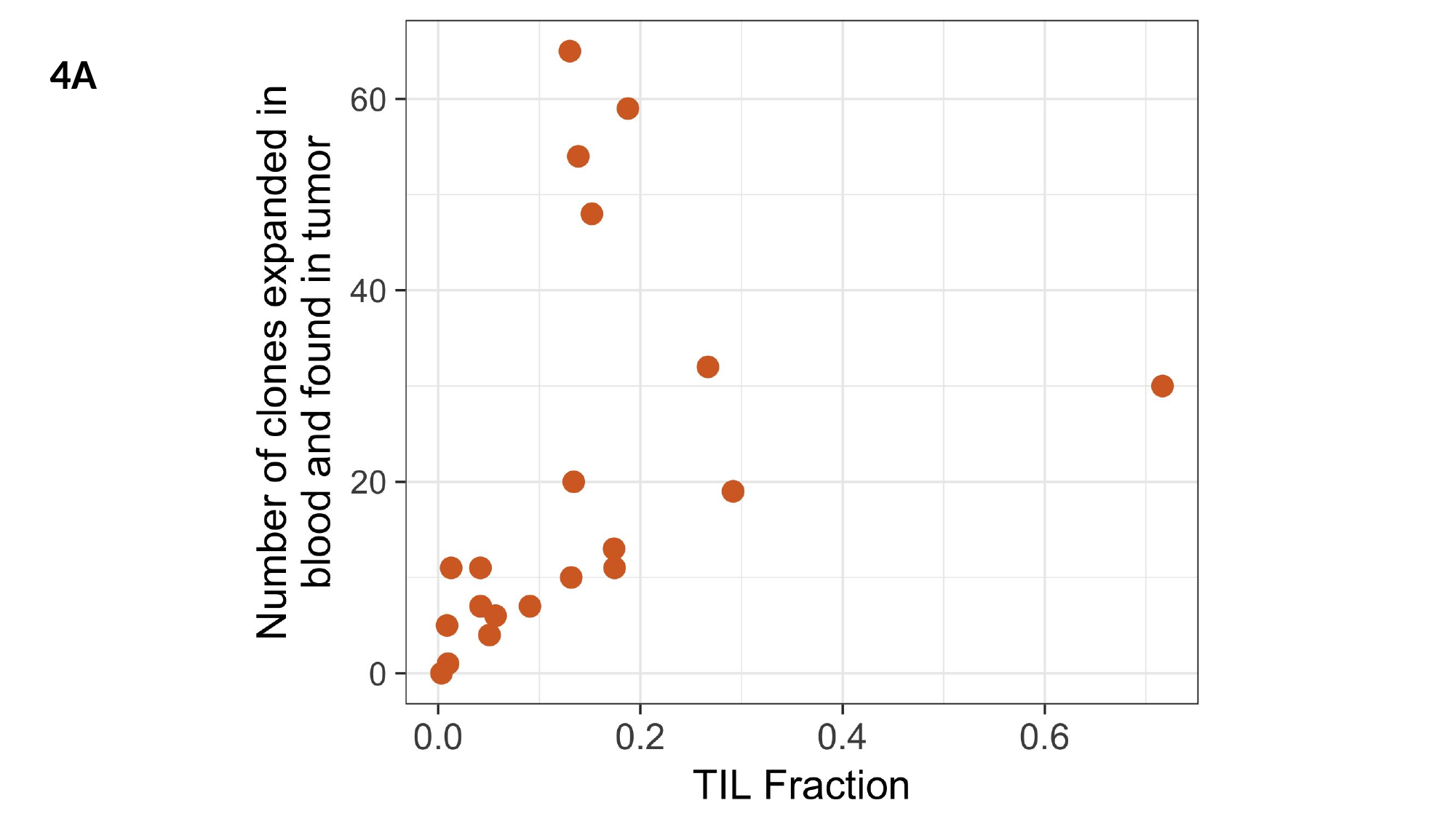

4A

## Slide 3
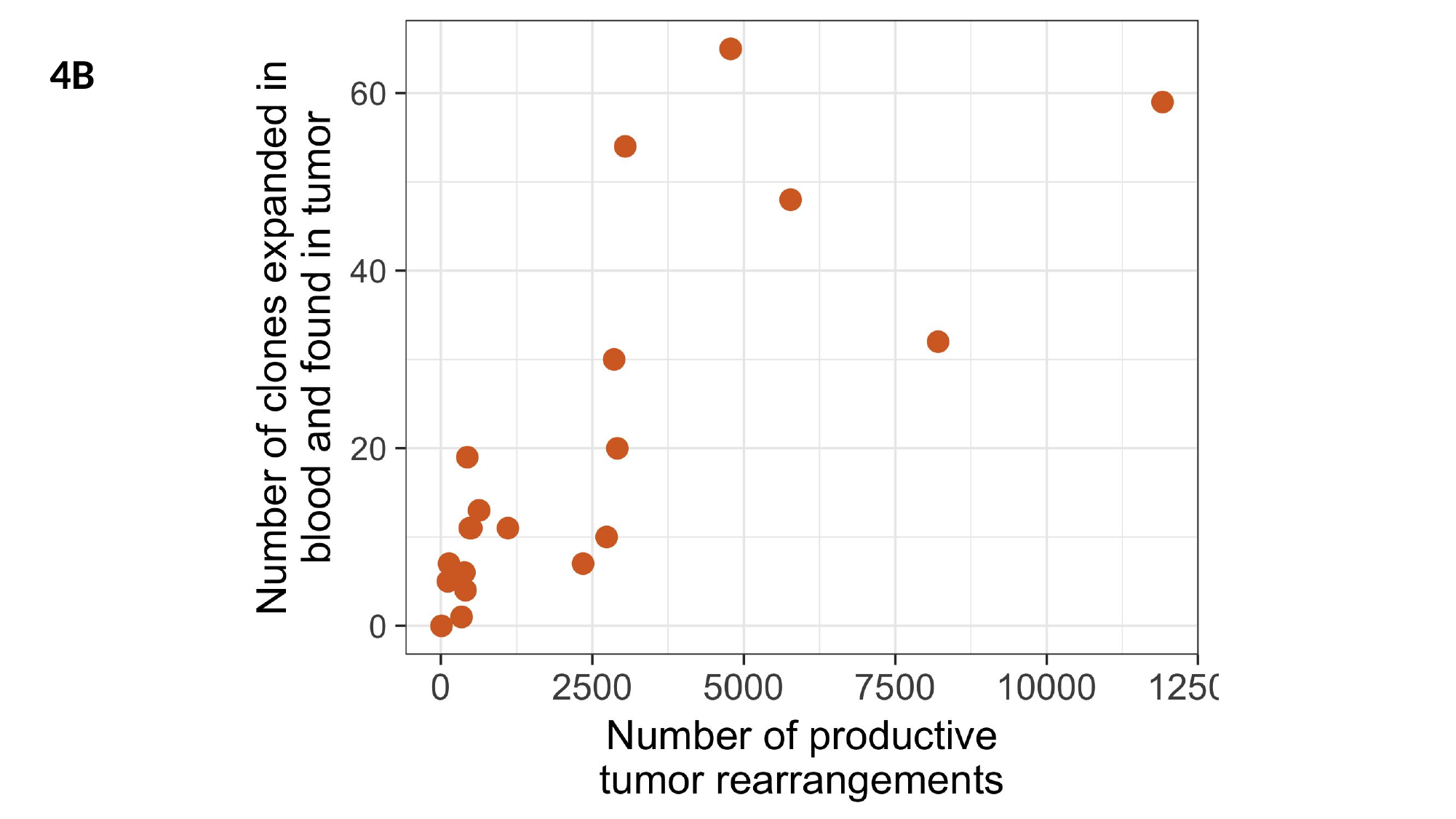

4B

Supplement: Supplementary file 1 — Figure S4. Clonal relatedness in the tumor and blood. The number of tumor-associated clones in the baseline tumor biopsies that were expanded in blood post-treatment was strongly correlated with both (A) tumor infiltrating lymphocyte (TIL) fraction (Rho 0.7299, p = 0.0003) and (B) TIL clone diversity (Rho 0.882, p = 2.7− 7). (PPTX 338 kb) [file 40425_2018_428_MOESM1_ESM.pptx]
